# Cell-Free Mitochondrial DNA in the CSF: A Potential Prognostic Biomarker of Anti-NMDAR Encephalitis

**DOI:** 10.3389/fimmu.2019.00103

**Published:** 2019-02-06

**Authors:** Yu Peng, Dong Zheng, Xiaomei Zhang, Suyue Pan, Teng Ji, Jun Zhang, Hai-Ying Shen, Hong-Hao Wang

**Affiliations:** ^1^Department of Neurology, Nanfang Hospital, Southern Medical University, Guangzhou, China; ^2^Department of Neurology, the Affiliated Brain Hospital of Guangzhou Medical University, Guangzhou, China; ^3^Department of Neurology, Randall Children's Hospital, Legacy Health, Portland, OR, United States; ^4^Department of Surgical Oncology, MD Anderson Cancer Center, University of Texas, Houston, TX, United States; ^5^Robert Stone Dow Neurobiology Laboratories, Legacy Research Institute, Legacy Health, Portland, OR, United States

**Keywords:** anti-NMDAR encephalitis, cerebrospinal fluid, cell-free mitochondrial DNA, cytokines, IL-6, IL-10, TNF-α, modified rankin scale

## Abstract

Anti-N-methyl-D-aspartate receptor (NMDAR) encephalitis is an autoimmune inflammatory brain disease that can develop a variety of neuropsychiatric presentations. However, the underlying nature of its inflammatory neuronal injury remains unclear. Mitochondrial DNA (mtDNA) is recently regarded as a damage-associated molecular pattern molecule (DAMP) that can initiate an inflammatory response. In the presenting study, we aimed to evaluate the levels of cell-free mtDNA in cerebrospinal fluid (CSF) of patients with anti-NMDAR encephalitis and to determine a potential role of cell-free mtDNA in the prognosis of anti-NMDAR encephalitis. A total of 33 patients with NMDAR encephalitis and 17 patients with other non-inflammatory disorders as controls were included in this study. The CSF levels of cell-free mtDNA were measured by quantitative polymerase chain reaction (qPCR). Cytokines including interleukin (IL)-6, IL-10, and tumor necrosis factor alpha (TNF-α) were measured by ELISA. The modified Rankin scale (mRS) score was evaluated for neurologic disabilities. Our data showed that the CSF levels of cell-free mtDNA and inflammation-associated cytokines were significantly higher in the patients with anti-NMDAR encephalitis compared with those in controls. Positive correlations were detected between the CSF levels of cell-free mtDNA and mRS scores of patients with anti-NMDAR encephalitis at both their admission and 6-month follow up. These findings suggest that the CSF level of cell-free mtDNA reflects the underlying neuroinflammatory process in patients with anti-NMDAR encephalitis and correlates with their clinical mRS scores. Therefore, cell-free mtDNA may be a potential prognostic biomarker for anti-NMDAR encephalitis.

## Introduction

Anti-N-methyl-D-aspartate receptor (NMDAR) encephalitis is a newly recognized acute autoimmune encephalitis caused by anti-neuronal autoantibodies, which affects mainly children and young adult females (1, 2). Its typical clinical manifestations include a progressive development of neurologic and psychiatric symptoms, including abnormal movements, seizures, impaired memory, and behavior disorders (2, 3). Anti-NMDAR encephalitis is usually associated with teratoma and often secondary to CNS infection caused by viruses or parasites (4, 5). Some studies have suggested that anti-NMDAR antibodies initiate the innate immune response and promote sterile neuroinflammation which leads to the lesioning of brain tissues (6, 7). However, one of the major challenges in the management of anti-NMDAR encephalitis is to identify biomarkers with diagnostic and prognosis predicting value.

Mitochondrial DNA (mtDNA) is a damage-associated molecular pattern molecule (DAMP) that can trigger activation of the human innate immune system and initiate an inflammatory response (8, 9). Usually, the basal level of CSF cell-free mtDNA stays low and reflects the normal turnover of mtDNA in the brain, whereas elevated CSF levels of mtDNA were reported in patients with traumatic brain injury and multiple sclerosis. This indicates that mtDNA plays a mediatory role in sterile inflammatory responses (10, 11). Thus, it is crucial to evaluate the profile of cell-free mtDNA in anti-NMDAR encephalitis and its potential relationship with the pathophysiology of this disease. In this study, we investigated the cell-free mtDNA profile in the CSF of patients with anti-NMDAR encephalitis vs. other non-inflammatory neurological disorders including peripheral nerve disease and hysteria. We further examined possible associations of their cell-free mtDNA profiles with the modified Rankin Scale (mRS) score and their cytokine profiles, including IL-6, IL-10, and TNF-α (12).

## Materials and Methods

### Patients and Controls

We recruited a total of 33 patients from the Department of Neurology, Nanfang Hospital, Southern Medical University, according to inclusion criteria based on the revised anti-NMDAR encephalitis diagnosis criteria of Graus et al. published in 2016 (2, 13). Specifically, all anti-NMDAR encephalitis patients were treatment-naïve, and their CSF was sampled at acute onset with confirmation of positive antibodies against the NR1 subunit of the NMDAR by cell-based analysis and negative for viral DNA and other pathogens. All the patients' CSF were negative for the detection of tuberculosis, cryptococcus, and some common viruses by PCR. All patients with anti-NMDAR encephalitis were treated with first-line treatment or combined second-line treatment (1). The control group consisted of a cohort of 17 age- and gender-matched patients with confirmed other non-inflammatory neurological disorders. Patients were tested negative for CSF antibodies against the NR1 subunit of the NMDAR. This study was conducted with the approval (NFEC-2018-095) of the Ethics Committee of Nanfang Hospital, Southern Medical University. Written informed consent was obtained from all patients for this study.

### CSF Collection and DNA Preparation

All 33 patients and the 17 control individuals were subjected to lumbar puncture for CSF analysis within 3 days of their admission; 15 of the 33 patients with anti-NMDAR encephalitis received another lumbar puncture for CSF re-evaluation at 6 months follow-up after discharge. CSF samples were processed within 30 min of collection and centrifuged at 1,000 g for 10 min. The CSF supernatants were then transferred to polypropylene tubes and stored at −80°C. DNA was extracted from supernatants using the QIAmp DNA Mini Kit (Qiagen GmbH).

### Quantitation of CSF Cell-Free mtDNA

The assessment of concentration of mtDNA was performed by quantitative polymerase chain reaction (qPCR) (Supplementary Material) (11). Each measurement consisted of biological duplicates and technical triplicates; the samples from anti-NMDAR encephalitis patients and control individuals were randomized to avoid batch effects.

### Determination of Inflammatory Cytokine Levels

The levels of inflammation-associated cytokines were quantified using Sandwich ELISA kits [Bender MedSystems GmbH (IL-6 and IL-10) (Vienna, Austria) and Cusabio (TNF-α) (Wuhan, China)] according to the manufacturers' instructions (Supplementary Material).

### Evaluation of mRS

The mRS score was evaluated for neurologic disabilities (14). All 33 patients with anti-NMDAR encephalitis were evaluated for mRS scores at the times of their admission, while 15 patients received re-evaluation for mRS scores at their 6-month follow-up.

### Statistical Analysis

Data were expressed as mean ± SD or the median (range). Statistical analyses were performed using SPSS version 20.0 (IBM Corp, Armonk, NY, USA). Independent-samples non-parametric tests were performed to compare the levels of CSF cell-free mtDNA or inflammatory cytokines between patients and controls. Paired *t*-tests were performed to compare parameters in the 15 patients at their admission vs. follow-up. Correlations among the quantitative parameters were evaluated using Pearson's test; correlations between mRS scores and quantitative parameters were assessed with Spearman's test. A *p* < 0.05 was regarded as statistically significant.

## Results

### Demographic and Clinical Features of Anti-NMDAR Encephalitis Patients

The demographic data and clinical features of patients (*n* = 33) and controls (*n* = 17) are shown in Table 1. All patients were confirmed by positive detection of anti-NMDAR autoantibodies in their CSF. Psychiatric symptoms (85%), electroencephalogram (EEG) abnormality (76%), and seizure onset (64%) were the most common clinical presentations in the patients with anti-NMDAR encephalitis. The other symptoms include fever, autonomic disturbances, disturbance of consciousness, abnormal movements, and so on. These patients with fever were neither identified with clues of bacterial infection nor increased levels of C reactive protein or procalcitonin. Notably, compared with their peak mRS scores at admission, the mRS scores at 6-month follow up in the 15 follow-up patients were significantly lower (paired *t*-test, *p* < 0.001), indicating the effectiveness of treatment.

**Table 1:** Clinic manifestations and characteristics of anti-NMDAR encephalitis and controls.

	**NMDAR (*n* = 33)**	**Control (*n* = 17)**
Gender (male/female)	14/19	8/9
Age (years, mean)	34.8 ± 17.6	35.0 ± 15.3
**PSYCHIATRIC AND NEUROLOGIC SYMPTOMS**		
Fever	19 (58%)	–
Disorders of memory, behavior, and cognition	28 (85%)	–
Seizures	21 (64%)	–
Autonomic disturbances	13 (39%)	–
Disturbance of consciousness	20 (61%)	–
Abnormal movements	16 (48%)	–
Abnormal electroencephalogram	25 (76%)	–
Ovarian teratoma	2 (6%)	–
**CSF ASSESSMENT**		
White blood cell count( × 10^6^/L, median)	3 (0, 17.5)	0 (0, 0)^**^
Protein (g/L, median)	0.28 (0.16, 0.58)	0.32 (0.21, 0.38)
IL-6(pg/ml, median)	7.30 (3.85, 13.40)	2.90 (2.60, 3.85)^***^
IL-10(pg/ml, median)	4.91 (3.64, 5.79)	0.91 (0.00, 1.13)^***^
TNF-α(pg/ml, median)	6.03 (4.33, 11.34)	1.23 (0.19, 2.30)^***^
**mRS SCORES**		
Maximum mRS scores	4 (4, 5)	–
6-months mRS scores	3 (2, 3.5)	–
Anti-NMDAR antibody	33	0

** p < 0.01;

*** *p < 0.001*.

### Cell-Free mtDNA and Inflammatory Cytokines in the CSF of Anti-NMDAR Encephalitis Patients

**Figure 1:**
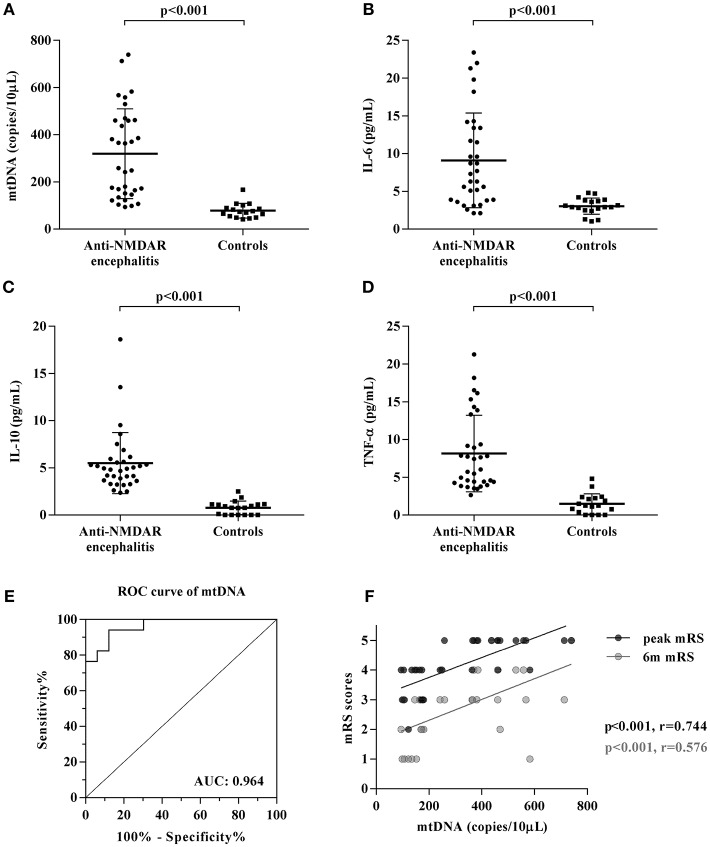
Changes of CSF mtDNA and inflammatory cytokines in anti-NMDAR encephalitis**. (A)** The q-PCR assessment of CSF levels of mtDNA showed elevated cell-free mtDNA copies in patients with anti-NMDAR encephalitis (NMDAR) vs. controls (CTL), while the levels of inflammation-related cytokines, IL-6 **(B)**, IL-10 **(C)**, and TNF-α **(D)** were also significantly changed in anti-NMDAR encephalitis. The *p*-values were indicated within figures. ROC analysis showed that when the cut-off value was set as 91.55 copies/10 μL, the sensitivity of CSF cell-free mtDNA test was 100% and the specificity was 76.5%, and the area under ROC curve (AUC^ROC^) was 0.964 (95% CI: 0.919–1.010, *p* < 0.0001) **(E)**. Potential correlations were analyzed between the patient' mRS scores acquired at their peak presentations (peak mRS) and 6-month follow-up (6 m mRS) to their corresponding CSF levels of cell-free mtDNA **(F)**.

To investigate the role of cell-free mtDNA in anti-NMDAR encephalitis, we evaluated the CSF levels of cell-free mtDNA in these patients (*n* = 33) and controls (*n* = 17) using a qPCR assay. As shown in Figure 1A, the copy number of CSF cell-free mtDNA was significantly elevated in anti-NMDAR encephalitis patients at the acute stage (258.2 copies/10 μl: 148.7, 461.3) compared to controls (73.6 copies/10 μl; 51.8, 95.1) (*p* < 0.001). To further evaluate the role of humoral immunity in anti-NMDAR encephalitis, we measured the CSF levels of inflammation-related cytokines, IL-6, IL-10, and TNF-α by ELISA. The ELISA data showed that the levels of pro-inflammatory cytokines IL-6 and TNF-α, as well as the anti-inflammatory cytokine IL-10, were significantly higher in patients with anti-NMDAR encephalitis compared with controls (*p* < 0.001, <0.001, <0.001, respectively) (Table 1, Figures 1B–D).

Furthermore, the receiver operating characteristic (ROC) curve analysis of cell-free mtDNA as a diagnostic biomarker of anti-NMDAR encephalitis showed that the sensitivity was 100% and the specificity was 76.5% when 91.55 copies/10 μl was selected as the cut-off value. Area under the ROC curve (AUC^ROC^) was 0.964 (95% CI: 0.919–1.010, *p* < 0.001) (Figure 1E).

### Altered CSF Levels of Cell-Free mtDNA and Inflammatory Cytokines in the Course of the Disease

The levels of CSF cell-free mtDNA in patients dropped significantly from 318.6 ± 196.7 copies/10 μl (peak) to 212.2 ± 129.8 copies/10 μl at 6-month follow up (*p* = 0.003), while the latter was still higher compared to controls (*p* < 0.001). The levels of CSF IL-6, IL-10, and TNF-α were also significantly reduced at 6-month follow up compared to that at the acute stage of anti-NMDAR encephalitis (*p* = 0.005, 0.003, <0.001, respectively).

### The Relationship Between CSF Levels of Cell-Free mtDNA and Inflammatory Cytokines in Anti-NMDAR Encephalitis

Correlation analysis was performed to demonstrate changes in the DAMP molecule, cell-free mtDNA with humoral immunity sponsors, e.g., IL-6, IL-10, and TNF-α. The analysis showed that there was a statistically significant correlation of CSF cell-free mtDNA with IL-6 (*r* = 0.307, *p* = 0.041), but neither with IL-10 (*r* = −0.085, *p* = 0.319) nor TNF-α (*r* = −0.050, *p* = 0.391) in the acute stage of anti-NMDAR encephalitis.

### Relationship Between CSF Levels of Cell-Free mtDNA and Clinical Outcome in Anti-NMDAR Encephalitis

The relationships between clinical outcome, mRS scores, and CSF levels of cell-free mtDNA, IL-6, IL-10, and TNF-α in patients with anti-NMDAR encephalitis were evaluated. There were significant positive correlations between CSF levels of cell-free mtDNA and mRS scores at both the acute stage (*r* = 0.744, *p* = 0.000) and the 6-month follow up (*r* = 0.576, *p* = 0.000, Figure 1F). However, no significant correlation was shown between the CSF levels of cell-free mtDNA and three cytokines IL-6, IL-10, and TNF-α, or clinical outcome.

## Discussion

In this cross-sectional study, we report for the first time the identification of cell-free mtDNA as a potential biomarker of mitochondrial damage in anti-NMDAR encephalitis. Our data demonstrating significantly increased CSF cell-free mtDNA suggests that cell-free mtDNA may participate in the pathogenesis of this disease, which in turn could reflect the severity of neurological impairment.

Both the innate and acquired immune system play a role in the pathogenesis of anti-NMDAR encephalitis (6). The innate immune system recognizes endogenous damage-associated molecular pattern (DAMP) molecules and exogenous pathogen-associated molecular pattern (PAMP) molecules, which will initiate a non-infectious or a pathogen-induced inflammatory response, correspondingly (15). Since mitochondria are evolutionarily derived from bacteria by endosymbiosis and therefore bear bacterial molecular motifs (15), mtDNA is considered an activator of the innate immune system. The mtDNA is a DAMP molecule that can initiate antimicrobial responses and inflammatory pathology (16) in various situations, such as shock, injury, infection, and cancer (15, 17–19). Cell-free mtDNA can be recognized by pattern-recognition receptors (PRRs), usually the toll-like receptor-9 (TLR-9) (8, 15) and result in increased expressions of inflammatory cytokines and pro-inflammatory molecules (9, 18, 20).

The anti-inflammatory cytokine IL-6 is increased in neuronal and CNS autoimmune diseases, such as neuromyelitis optica and multiple sclerosis (21, 22). Mechanistically, IL-6 stimulates B-cell differentiation, promotes the survival of plasmablasts, and enhances the intrathecal production of anti-NMDAR antibodies (12, 23, 24). Here, we demonstrated that the CSF level of IL-6 was significantly increased and positively correlated with CSF mtDNA levels in anti-NMDAR encephalitis. However, the other two inflammatory cytokines, IL-10 and TNF-α lack correlation with changes of CSF mtDNA though they also increased in the patients with anti-NMDAR encephalitis. These findings suggest that while cytokines may play an essential role in the pathogenesis of anti-NMDAR encephalitis, IL-6 may specifically act as a downstream molecule of the mtDNA mediated immune response.

Since a positive prognosis of anti-NMDAR encephalitis is closely linked to early treatment, early diagnosis of this disease is crucial. CSF biomarkers that have a strong correlation with the prognosis of anti-NMDAR encephalitis would be particularly useful in the assessment of the severity and evaluation of recovery for patients. In this study, we demonstrated that the CSF levels of cell-free mtDNA positively correlated with both the maximum and 6-month follow up mRS scores. This suggests that a higher level of CSF cell-free mtDNA may indicate a more severe clinical presentation and worse prognosis of anti-NMDAR encephalitis. Therefore, changes in CSF cell-free mtDNA, combined with changes in anti-NMDAR antibody titer, may become a promising indicator for prognosis predicting and monitoring in anti-NMDAR encephalitis.

CSF cytotoxicity has been thought to be a cause of certain neurodegenerative diseases such as amyotrophic lateral sclerosis (ALS) (25). Transfering the CSF of ALS pathients to rat cerebral ventricle could provoke changes similar to those found in the disease (26). Though the pathogenesis of anti-NMDAR encephalitis is not all clear, tumor is though to be associated with anti-NMDAR encephalitis. CNS infections were also thought as immunological triggers in some cases, especially in relapse post-herpes simplex virus encephalitis (4, 27). In the present study, all the patients' CSF were negative for the detection of tuberculosis, cryptococcus, and some common viruses by PCR. The correlation between mitochondrial DNA and infection needs to be clarified in further studies.

Yet, this result has some limitations: the control group comprised subjects with non-inflammatory CNS diseases. It may be better to include a control cohort with viral encephalitis since it is initiated by PAMP, which usually mimics presentations of anti-NMDAR encephalitis. Future studies should include a control group of subjects with PAMP related encephalitis.

## Conclusion

This report details, for the first time, the role of CSF cell-free mtDNA in anti-NMDAR encephalitis. Our findings suggest that CSF cell-free mtDNA may be implicated in the pathogenesis of anti-NMDAR encephalitis. In addition, CSF cell-free mtDNA may also act as a prognostic biomarker as it correlated positively with mRS scores and indicated that higher levels of CSF cell-free mtDNA were associated with more severe clinical presentations and a worse prognosis.

## Availability of Data and Material

We declare that materials described in this manuscript, including all relevant raw data, will be freely available to any scientist wishing to use them for non-commercial purposes, without breaching participants' confidentiality.

## Author Contributions

H-HW, H-YS, TJ, and JZ co-conceived this study and designed the experiments. YP, SP, DZ, and XZ collected the CSF samples and clinical data. YP, DZ, and H-HW performed the experiments and analyzed the data. YP, H-HW, and H-YS wrote the manuscript and prepared the table, figures. YP, JZ, and H-HW revised the article. All authors read and approved the final manuscript and agreed to submit it for publication.

### Conflict of Interest Statement

The authors declare that the research was conducted in the absence of any commercial or financial relationships that could be construed as a potential conflict of interest.
