# Supplementary material for: Cell-Free Mitochondrial DNA in the CSF: A Potential Prognostic Biomarker of Anti-NMDAR Encephalitis
Source: Front Immunol. 2019 Feb 6;10:103. doi: 10.3389/fimmu.2019.00103 (PMC6375341; doi:10.3389/fimmu.2019.00103)
Supplement: Supplementary file 1 [file Data_Sheet_1.docx]

**Supplementary Material**

**Quantitation of copy number of CSF cell-free mtDNA**

Referring to Varhaug, et al. (1), we filtered the CSF samples by using Amicon Ultra 0.5 ml, 30kDa, columns. Then, extracted DNA from 200µl CSF using the QIAmp® DNA Mini Kit (Qiagen GmbH) per manufacturer’s guidelines. The concentration of mtDNA was determined by quantitative polymerase chain reaction (qPCR). MTND1 gene was used to quantify mtDNA and the nuclear encoded gene amyloid precursor protein (APP) to detect any nuclear DNA contamination. To ensure that we only measured cell-free mtDNA, samples showing detectable amplification of APP were discarded. The amount of mtDNA was calculated using a standard curve derived from serial dilutions of a gel-purified MTND1 amplicon. The serial dilutions contained 10^1^-10^8^ copies/µl. The following primers, probes and conditions were used. MTND1: forward primer: 5’-CCCTAAAACCCGCCACATCT-3’, reverse primer: 5’-GAGCGATGGTGAGAGCTAAGGT-3’, probe: 5’-FAM-CCATCACCCTCTACATCACCGCCC-3’. APP: forward primer: 5’-TGTGTGCTCTCCCAGGTCTA-3’, reverse primer: 5’-CAGTTCTGGATGGTCACTGG-3’, probe: 5’-VIC-CCCTGAACTGCAGATCACCAATGTGGTAG-3’. 10µl CSF samples were used per PCR reaction. All standard curves had amplification efficiency over 90%. PCR was performed in triplicate using an ABI 7500 Fast Real-time PCR system (Life Technologies Corporation) using TaqMan ® Fast Advanced Master Mix (Thermofisher). Thermal cycling consisted of the following profile: one cycle at 95 °C for 20 seconds, 45 cycles at 95 °C for 3 seconds and 60 °C for 30 seconds. Each run contained a negative control for both MTND1 and APP. To ensure reproducibility, 10 of the samples were replicated, with results within a 5% coefficient of variation range. To avoid batch effect a random mix of controls and patient samples were always run together.

**Determination the levels of inflammatory cytokines: IL-6, IL-10, and TNF-α.**

The ELISA kits of IL-6, IL-10 and TNF-α were used according to the manufactory protocols. 50μL of standards, controls or CSF samples were added with 50μL of sample diluent to the plate, then 50 µl of Biotin-Conjugate was added to wells and incubated 2h at 25°C (IL-6, IL-10)/37°C (TNF-α) with shaking. The wash step was repeated four times. 100 μl of diluted Streptavidin-HRP was added to all wells and incubated for 1 h. Lastly, after 4 times of wash, 100 μL of TMB (Supersenitive 3, 3′, 5, 5′ tetramethylbenzidine) was added and the plates were incubated for 10min at 25°C. Direct exposure to intense light was avoided. The enzyme reaction was stopped by quickly pipetting 100μl of Stop Solution into each well. Absorbencies were determined photometrically at 450 nm using an ELISA plate reader for concentration calculations.

**Reference:**

1. Varhaug KN, Vedeler CA, Myhr KM, Aarseth JH, Tzoulis C and Bindoff LA. Increased levels of cell-free mitochondrial DNA in the cerebrospinal fluid of patients with multiple sclerosis. *Mitochondrion* (2017) 34:32-35. doi:10.1016/j.mito.2016.12.003
